# Impacts of Plastics on Plant Development: Recent Advances and Future Research Directions

**DOI:** 10.3390/plants12183282

**Published:** 2023-09-15

**Authors:** Enikő Mészáros, Attila Bodor, Etelka Kovács, Sarolta Papp, Kamilla Kovács, Katalin Perei, Gábor Feigl

**Affiliations:** 1 Department of Plant Biology, University of Szeged, Közép fasor 52, H6726 Szeged, Hungary; 2 Department of Biotechnology, University of Szeged, Közép fasor 52, H6726 Szeged, Hungary; bodora@bio.u-szeged.hu (A.B.); perei@brc.hu (K.P.); 3 Institute of Biophysics, Biological Research Centre, Temesvári krt. 62, H6726 Szeged, Hungary

**Keywords:** plastic, microplastic, toxicity, plants

## Abstract

Plastics have inundated the world, with microplastics (MPs) being small particles, less than 5 mm in size, originating from various sources. They pervade ecosystems such as freshwater and marine environments, soils, and the atmosphere. MPs, due to their small size and strong adsorption capacity, pose a threat to plants by inhibiting seed germination, root elongation, and nutrient absorption. The accumulation of MPs induces oxidative stress, cytotoxicity, and genotoxicity in plants, which also impacts plant development, mineral nutrition, photosynthesis, toxic accumulation, and metabolite production in plant tissues. Furthermore, roots can absorb nanoplastics (NPs), which are then distributed to stems, leaves, and fruits. As MPs and NPs harm organisms and ecosystems, they raise concerns about physical damage and toxic effects on animals, and the potential impact on human health via food webs. Understanding the environmental fate and effects of MPs is essential, along with strategies to reduce their release and mitigate consequences. However, a full understanding of the effects of different plastics, whether traditional or biodegradable, on plant development is yet to be achieved. This review offers an up-to-date overview of the latest known effects of plastics on plants.

## 1. Introduction

The planet boundary concept defines the limits that humanity must not exceed in order to not endanger the favorable conditions in which it has been able to develop and live sustainably in a safe ecosystem [1]. In 2009, nine specific boundaries were established, including climate change, the loss of biosphere integrity, the disruption of nitrogen and phosphorus biogeochemical cycles, land use changes, ocean acidification, freshwater consumption, stratospheric ozone depletion, increasing aerosols in the atmosphere, and chemical pollution [2]. The last one encompasses the release of novel entities such as synthetic organic pollutants, heavy metal compounds, and plastic pollution into the environment [2].

After a temporary halt due to the COVID-19 pandemic in 2020, global plastic production rose to 390.7 million tons in 2022 [3], with projections indicating that its usage will reach 1231 million tons by 2060 [4]. Fossil-based plastics accounted for 90.2% of the world’s production in 2021, while bio-based/bio-attributed plastics and post-consumer recycled plastics comprised 8.3% and 1.5% of global plastic production, respectively [4]. In China, the total production of plastic products exceeded one billion tons by the end of 2019, establishing itself as the world’s largest producer and consumer of plastic [5]. In 2021, China was responsible for almost one third of the world’s plastic production (32%), followed by North America with 18%, then the rest of Asia with 17%, Europe with 15%, and Africa and the Middle East with 8% [4].

Plastics are artificially produced and polymerized from various monomers [6]. The most commonly found plastics in the environment are polyethylene (PE), polyvinyl chloride (PVC), polypropylene (PP), polyethylene terephthalate (PET), and polystyrene (PS) [7]. These materials have a wide range of applications, including, but not limited to, smartphones, food packaging, and 3D printing, which stems from its design adaptability, affordability, ability to be shaped, lightweight nature, and biologically inert properties [8]. Consequently, plastic has the advantage of replacing traditional materials, such as paper, wood, or metal.

Once released into the environment, plastics pose a significant threat, due to their slow decomposition, which can take hundreds of years. The primary sources of plastic pollution are the fragmentation of larger plastic items, such as bags, bottles, and packaging materials. Over time, these items break down into smaller pieces, eventually becoming macroplastics (>2.5 cm), microplastics (<5 mm, MPs), or even nanoplastics (<100 nm or <1000 nm) [9,10]. MPs have two main sources: primary and secondary. The primary sources include drugs, paints, cosmetics, biomedical equipment, and other items, while the secondary sources refer to the mechanical, thermal, and biological degradation of macroplastics [11]. Other sources of MP pollution include the use of synthetic textiles, such as polyester and nylon, which shed microfibers during washing, and the use of cosmetics or personal care products that contain microbeads. Additionally, MPs can be released into the environment through the disposal of electronic waste, such as mobile phones and computers [12]. MPs can interact with other chemicals, leading to the accumulation of organic and inorganic pollutants on their surfaces [13]. Polychlorinated biphenyls (PCBs), polycyclic aromatic hydrocarbons (PAHs), antibiotics, and other chemicals pose a significant concern when they attach to MPs [13]. Consequently, MPs are categorized as emerging persistent pollutants that occur widely in various ecosystems.

Most plastic becomes waste shortly after use, and, therefore, a significant amount of plastic is constantly released into the environment. According to estimations, about 12.7 million tons of plastic has ended up in the marine environment so far [14], and MPs have been detected in the air [15] and in animals, where they can accumulate in various tissues, posing a long-term threat. Moreover, MPs can be transferred through the trophic food chain, reaching final consumers, including predators and humans [16]. Importantly, recent research has revealed the presence of MPs in human blood, with a measured total amount of 1.6 µg/L. The main compounds found were PET, PE, and PS. These results show that these particles are bioavailable and can be absorbed into the bloodstream of humans [17].

Considering the persistence and widespread distribution of MPs in the soil, it is necessary to recognize their potential impacts on terrestrial plants. Based on estimations, approximately 32% of all plastic produced ends up in the soil [18]; therefore, soils can be a much larger sink for plastics than salt water and freshwater [19]. Consequently, the presence of plastics in soil ecosystems will have an impact on the organisms that live there, including plants.

Exploring the relationship between plants and plastics is currently becoming a hot research topic; however, despite the growing body of published results, it is still challenging to draw definitive conclusions. After reviewing the available literature, it seems to be evident that the impacts of MPs on higher plants depend on various factors, such as the properties of the MPs, the specific plant species involved, and the surrounding environmental conditions. Under certain experimental conditions, MPs have induced no effects, or even positive effects, on higher plants. However, a larger body of evidence demonstrates that MPs can directly and indirectly impede the growth of higher plants. As a recently published paper [20] has already compiled the known effects of plastics on plants, this article aims to summarize the most recent experimental data, with a primary focus on the effects of plastics on the rhizosphere, particularly on plants.

## 2. Impact of Plastics on Soil, Plants, and Ecosystems

Soil, as the uppermost loose, fertile layer of the Earth’s surface, is essential for sustaining life. It provides plants (and thus animals and humans) with nutrients and water, while also storing and transforming materials [21]. To safeguard the healthy status of soil, models show that the concentration of MPs should not exceed the range of 2128–14,435 mg for each kg soil in order to keep up with half of the currently present soil biota or soil properties [19]. However, agricultural soil serves as a larger sink for MPs compared to water [22]. MPs can enter soils through various pathways, including sewage and sludge irrigation and residual mulching film decomposition [23,24]. An increased amount of MPs in the soil can directly alter the soil physicochemical properties, leading to reduced soil aeration, increased erosion, altered soil pH, and reduced nutrient availability for plants, and, hence, lower crop yields [25,26]. Additionally, the presence of MPs may alter the composition of soil microbial communities, reducing the diversity and abundance of beneficial microorganisms, which play essential roles in soil fertility and plant growth. Furthermore, MPs can act as carriers of other chemical contaminants in the soil, causing damage to plant and human health, when they reach the food chain [13,18,19].

Once in the soil, some MPs can be transported by external factors and enter surface water and groundwater via horizontal and vertical migration, while others are absorbed or accumulated by plants and soil-dwelling organisms [27]. In addition to the adverse effects of MPs on soil properties [12], several studies have also confirmed the extensive negative impact of MP accumulation on soil biology [28]. For example, MPs can disrupt the functional and structural diversity of soil microbial communities [29] and harm soil-dwelling animals, plants, and microorganisms by damaging DNA, inducing oxidative stress, and impairing metabolic processes [30,31]. However, these effects are often contradictory, and vary depending on factors such as MP shape and size, polymer type, degradability, or the presence of additives and impurities [28].

When it comes to MP contamination in soil, invertebrates, particularly earthworms, have received significant attention in studies, as they are vital components of the soil food chain and can be used to assess the toxicity of contaminants such as MPs in soil [19]. Earthworms have the ability to ingest MPs and reduce their size, easing their decomposition [32]. Reduced growth rates [33], immunological stress responses [34], damaged intestinal cells and DNA [35], and increased mortality [36] are among the negative outcomes caused by exposure to MPs. In a recently published study investigating on the potential harm of low-density polyethylene (LDPE) to earthworms (*Eisenia fetida*), Mondal et al. [37] found that, during the 28-day exposure period, no mortality or weight loss relative to the controls was observed. However, they noticed damage to the surfaces of the earthworms’ skin caused by exposure to the LDPE MPs. The induced skin surface damage and MP uptake can adversely affect the growth and reproduction of *E. fetida* after long-term MP exposure.

Under the influence of environmental factors, MPs can be broken down into nanoplastics (NPs), which are plastic particles smaller than a micron in size [38]. Similar to MPs, NPs also come from a variety of sources, including the decomposition of plastic products in the environment and the influx of microbeads and raw materials used in industry [39]. Significant amounts of NPs are present in our daily lives, derived, for example, from tire wear, washing textiles, and using personal hygiene products [40,41,42]. The increasing abundance of NPs poses potential environmental hazards, adding to the global problem of environmental pollution. Living organisms can absorb NPs from the environment, triggering various stress responses. Since plants play an important role in ecosystems, the bioaccumulation of NPs can be an entry point for plastics into the food chain [43]; therefore, it is of utmost importance to study and explore the interactions and mechanisms between NPs and plant [38]. NPs can enter plants through various pathways, such as root or foliar uptake. The root uptake pathway can be significant for terrestrial plants, such as crops grown in agricultural fields, since they rely on soil water for their survival [10]. Foliar uptake occurs when plastic particles are present in the air, and the plant leaves absorb them through atmospheric deposition. This pathway can be significant for plants growing in urban environments or near industrial areas with elevated air pollution [44].

Plants are essential for our ecosystem, providing food, oxygen, and numerous other benefits, which can be jeopardized by anthropogenic stressors. In recent years, the issue of plastic pollution has emerged as a growing concern [45,46]. While plastic waste is known to harm marine life [47], scientists have only recently begun to realize the impact of MP pollution on terrestrial ecosystems [48] and its effect on plants [49]. The potential impacts of MPs and NPs on plants and the issue of food safety concerns for crops have been discussed recently [49], and subsequent studies have confirmed that NPs can be absorbed by plant roots and translocated to the aboveground aerial tissues [50]. These studies have demonstrated that MPs and NPs can induce physiological changes in plants, such as a reduction in growth, photosynthesis, and antioxidant activity, as well as alterations in gene expression and root exudate profiles [49,51].

Currently, most research focuses on the distribution and potential toxicity of MPs/NPs in aquatic ecosystems and their transmission to human food sources. However, there is also an urgent need to investigate the potential harmful effects of MPs/NPs on terrestrial plants and crop production. Although some recent studies, such as those by Zhou et al. [52], Yin et al. [53], Okeke et al. [54], and Yadav et al. [55], have explored the impacts of MPs on agroecosystems, vascular plants, food chains, and plant growth, these studies offer only a superficial analysis, and there is a lack of explanation regarding the underlying causes (such as the antagonistic effect of MPs/NPs on plants) that contribute to the dysfunction of the food chain. Therefore, it is crucial to conduct an in-depth analysis of the potential issues related to the entry of plastic particles into plants, the subsequent weakening of plant defense mechanisms, the putative factors determining the toxicity of MPs/NPs, and their interference with food quality and quantity. Moreover, as research in this area is still in its early stages, there is limited information on how to mitigate the adverse effects of MPs/NPs in plants.

## 3. Effects of Plastics on Plant Development

Studies have shown that plastics generally have a negative effect on plant development, which might manifest in alterations in both germination and root or shoot growth. These changes, however, depend on several factors, including the environmental conditions, plant species, and plastic concentration. Several types of plastics have been tested, including PS, PE, PVC, and biodegradable plastics, which are summarized below.

### 3.1. Effects of Polystyrene on Plant Development

PS is a widely used synthetic polymer derived from aromatic hydrocarbons called styrene monomers. With an annual production volume of several million tons, it ranks among the most commonly utilized plastics. Although PS is naturally transparent, it can be dyed for various applications. These applications include protective packaging containers, lids, bottles, trays, cups, and disposable cutlery [56].

The relationship between PS and plants has been studied in a range of applied concentrations and experimental systems, the vast majority of which have demonstrated growth inhibition. The studies dealing with the effects of polystyrene mentioned in the text are summarized in Table 1. 

In realistic field conditions, as stressors are seldom found in isolation within the environment, MPs and NPs can coexist with arsenic (As) in the soil, potentially causing toxic effects on plant growth and escalating the accumulation of arsenic in plants throughout the food chain. Two studies centered on the impact of PS plastic fragments on rice (*Oryza sativa* L.) plants, along with the absorption of As. The studies aimed to explore how MPs influence the overall As uptake in rice seedlings and the subsequent accumulation of As within the rice tissues. The growth responses were found to be dependent on the size of the NP/MP particles. Interestingly, the exposure of rice seedlings to MPs slightly mitigated the adverse effects of As on plant leaf growth and reduced root activity when compared to rice seedlings that were exposed to arsenic alone [57]. An additional study conducted by Xu et al. [58] revealed a noteworthy decline (*p* < 0.001) in aboveground tissue biomass due to As treatment, as compared to the control group. This effect was more pronounced when both MP and As stresses were combined. However, the root biomass exhibited only minor alterations. These results indicate that the combined effect of MPs and As on plants differs from the individual effects of MPs or As alone. Furthermore, other studies reported root and shoot inhibition in the presence of PS MPs on rice [59], which is consistent with earlier findings [60]. 

Lettuce *(Lactuca sativa* L.) is a major food source grown worldwide. Wang et al. [61] observed the effects of PS plastic on lettuce growth and found that PS MPs can disturb the antioxidant system, change the gene expression in roots, and influence the root exudate profiles. MP stress increased the expression of genes involved in different antioxidant systems at different times in the roots and leaves (ascorbic acid, terpenoids, flavonoids, and sphingolipids). The solutions with higher MP concentrations further inhibited lettuce growth compared to the controls, and the fresh leaf weight, plant height, and number of leaves were significantly reduced in all of the plants grown in the presence of MPs (*p* < 0.05) [61].

The response of tomato (*Lycopersicon esculentum* L.) plants to PS was investigated in plants grown in a hydroponic medium. Shi et al. [62] conducted an experiment with 13 treatment groups, including PS concentrations ranging from 0.1 to 1 mg/L, with tomato seedlings for 14 days. The results showed that PS MPs inhibit tomato plant growth and cause severe oxidative stress. Several treatments reduced the length of the shoot and root of the tomatoes and also affected some important metabolic pathways, including the tricarboxylic acid cycle and glutathione metabolism [62].

There are also some studies with mung bean (*Vigna radiata* L.) and onion (*Allium cepa* L.) plants in pot conditions. These studies have demonstrated that PS had a more negative effect on root growth in mung beans compared to onions. Biba et al. [63] reported that there were no significant changes in onion root growth after exposure to any of the tested PS MP concentrations, compared to the control, although the highest concentration (1 g/L) caused a slight decrease in length. On the other hand, Chen et al. [64] found that any applied concentration of PS MPs significantly reduced the growth characteristics of mung bean plants, including the shoot and root growth. PS also had a negative effect on the root and shoot growth of water spinach (*Ipomoea aquatica* Forssk.) and dandelion (*Taraxacum asiaticum* Dahlst) plants in hydroponic conditions but had no significant effect on their seed germination [65,66]. 

On the other hand, PS did not significantly reduce any root or shoot growth parameters in corn (*Zea mays* L.) plants [67], and no negative effects were detected in soybean (*Glycine max* L.) plants either [68]. Conversely, when watermilfoil (*Myriophyllum verticillatum* L.) was tested, PS MPs had a negative effect on shoot growth [69]. Interestingly, a recently conducted study showed that the root and shoot growth of corn plants were inhibited by PS [70]. Another experiment showed that PS had a negative effect on root growth in soybean plants, while no changes were observed in shoot growth [71]. However, a recent study found a slight effect on seed germination and sprout growth in soybean plants [68].

**Table 1:** The effect of polystyrene-based plastics on the development of plants. “−” represents inhibition, “0” marks no change, while “n.d.” marks parameters not determined.

Species	Concentration	Particle Size	Medium Used	Effect on			Notes	Reference
				Germination	Root Growth	Shoot Growth		
*Oryza sativa* L.	50 mg/L NPs/MPs and 250 μg/L As(III)/As (V)	82 and 200 nm	Hydroponic	n.d.	−	n.d.	NPs/MPs may coexist with As in soil and induce potentially toxic effects on the crop’s growth.	[57]
*O. sativa*	5 mg/mL	139 nm	Pot experiment	n.d.	−	−	Growth inhibition.	[58]
*O. sativa*	0, 0.5, 1.5, and 3.0 mg/L	10 ± 0.37 μm	Hydroponic	n.d.	−	−	Decline in growth, nutrient profile, perturbed gas exchange attributes, and enhanced oxidative damage.	[60]
*Lactuca sativa* L.	10, 20, 30, 40, and 50 mg/L	0.2 μm	Hydroponic	n.d.	−	−	Disturbing the antioxidant system and changing gene expression of the roots.	[61]
*Lycopersicon esculentum* L.	0.1 and 1 mg/L	5.23–17.21 μm	Hydroponic	n.d.	−	−	MPs caused severe oxidative stress.	[62]
*Allium cepa* L.	25, 50, and 100 μg/mL	n.d.	Pot experiment	n.d.	0	n.d.	The activation of antioxidant enzyme machinery of root cells successfully decomposed ROS and prevented oxidative damage.	[63]
*Vigna radiata* L.	2–4 mg/kg	5 µm	Pot experiment	n.d.	−	−	Perturbed rubisco activity and changed amino acid concentration in the plant tissues.	[64]
*Taraxacum asiaticum* Dahlst	1, 5, and 10 mg/L	80 nm	Hydroponic	n.d.	−	−	Inhibited the activities of rubisco and DHA by destroying the tertiary structure of the enzymes.	[65]
*Ipomoea aquatica* Forsk	0.5–10 mg/L	80 nm	Hydroponic	n.d.	−	−	The migration of PS NPs in roots, stems, and leaves was tracked.	[66]
*Zea mays* L.	10 mg/L	0.2–1.0 μm and 2.0 and 0.5 μm	Hydroponic	n.d.	0	0	The smaller-sized PS beads were absorbed by the roots.	[67]
*Glycine max* L.	80 μg/mL	20 nm and 1 µm	Hydroponic	0	0	n.d.	PS nanoparticles were distributed in the roots of bean sprouts more than in the stems and cotyledons.	[68]
*Myriophyllum verticillatum* L.	5 g/L	100 nm	Hydroponic	n.d.	n.d.	−	Induced changes in SOD activity, ROS production rate, and osmotic regulator content.	[69]
*Z. mays*	10 and 100 mg/L	25 nm	Pot experiment	n.d.	−	−	Polystyrene nanoplastics and Cd entered the root system through the stomatal pathway.	[70]
*G. max*	0, 12.5, 25, and 50 mg/L	20–30 nm	Petri dish experiments	n.d.	−	0	PS NPs affected growth and absorption of elements and the production of ROS and lipid peroxidation in the roots and leaves.	[71]

### 3.2. Effects of Polyethylene Plastics on Plant Development

PE is the most widely used plastic, mainly by the packaging industry. It has several types, such as high-density polyethylene (HDPE), medium-density polyethylene (MDPE), low-density polyethylene (LDPE), and cross-linked polyethylene (XLPE/PEX) [72].

The impact of PE on plant development has been explored in several recent studies under different conditions (Table 2). Numerous studies have shown that PE negatively affects both the root and shoot growth of corn plants [73,74,75]. Additionally, a significant reduction in root and shoot growth has been observed in cucumber (*Cucumis sativus* L.) and water moss (*Salvinia auriculata* Aubl.) plants in hydroponic experiments [76,77]. An experiment with lentil (*Lens culinaris* Medik) plants demonstrated that their germination was inhibited by PE [78]. Moreover, a study found that PE significantly increased the shoot growth of lettuce, barley (*Hordeum vulgare* L.), cucumber, and tomato (*Solanum lycopersicum* L.) plants [50,79]. Conversely, according to another study, root development was reduced by PE in cucumber and tomato plants [79].

The effect of HDPE on plants has also been researched. For instance, barley, sand couch-grass (*Thinopyrum junceum* L.), and ice plant (*Carpobrotus* sp.) plants are sensitive to HDPE MPs stress, as it inhibits their root and shoot growth [80,81].

### 3.3. Effects of Polyvinyl Chloride Plastics on Plant Development

PVC is a chemically inert material that can exist in both flexible and rigid forms. Rigid PVC is easily machinable, thermoformable, weldable, and can even be bonded using solvents. PVC can also be machined using standard metalworking tools and is less difficult to machine to tight tolerances and finishes. PVC resins are commonly blended with other additives, such as impact modifiers and stabilizers, to create a wide range of PVC-based materials [82].

Several recent studies have demonstrated the negative effects of PVC MPs on plant development in different growing media, which are presented in Table 3. PVC has been found to inhibit the growth of corn and plumed cockscomb (*Celosia argentea* L.) roots and shoots [83,84]. However, there was no significant effect observed in sweet potato (*Ipomoea batatas* L.) plants [85]. In duckweed (*Spirodela polyrhiza* L.) plants, PVC MPs were found to have a greater impact on root extension than shoot growth [86,87]. Moreover, Song et al. [88] examined the effects of PVC plastic fragments on rice and found a negative effect on root growth but no significant impact on shoot development.

### 3.4. Effects of Biodegradable Plastics on Plant Development 

Bioplastics are materials or products derived from biomass that are driving the evolution of plastics. These bio-based plastic products are typically derived from sources such as corn, sugarcane, or cellulose. They offer two main advantages over conventional products. Firstly, using biomass as a raw material saves fossil resources, as it is renewable and has the unique potential to be CO_2_ neutral. Additionally, certain types of bioplastics exhibit biodegradability as an additional property [89].

The effect of bio-based plastics on plant development has been examined in several studies recently (Table 4). Corn appears to be a particularly sensitive species to biodegradable plastics such as polybutylene adipate terephthalate (PBAT) and polylactic acid (PLA), as indicated by reduced root and shoot growth [73,74]. Poly(3-hydroxybutyrate-co-3-hydroxyvalerate) PHBV, a microbial biopolymer with excellent biocompatibility and biodegradability, has been shown to negatively impact corn root and shoot growth in soil [90]. Furthermore, pot experiments with basil (*Ocimum basilicum* L.), sand couch-grass, and ice plant plants have demonstrated that biodegradable plastics such as corn-starch-based bioplastic and starch-based polymers inhibited root and shoot growth [81,91]. In the case of sorghum (*Sorghum saccharatum* L.), garden cress (*Lepidium sativum* L.), and white mustard (*Sinapis alba* L.) plants, Liwarska-Bizukojc et al. [92] studied the effect of bio-based plastics such as PLA and polyhydroxybutyrate (PHB). The results indicated a significant reduction in both root and shoot growth. However, there were no statistically significant differences in germination efficiency between tests conducted with and without plastic particles in the soil. Interestingly, in red cherry tomato (*Lycopersicon esculentum* Mill.) and lettuce plants, plastic particles such as PBAT and PLA inhibited germination and shoot growth as well [93]. Additionally, PLA inhibited the root and shoot development of cucumber and barley plants [76,80].

## 4. Conclusions

**Figure 1:**
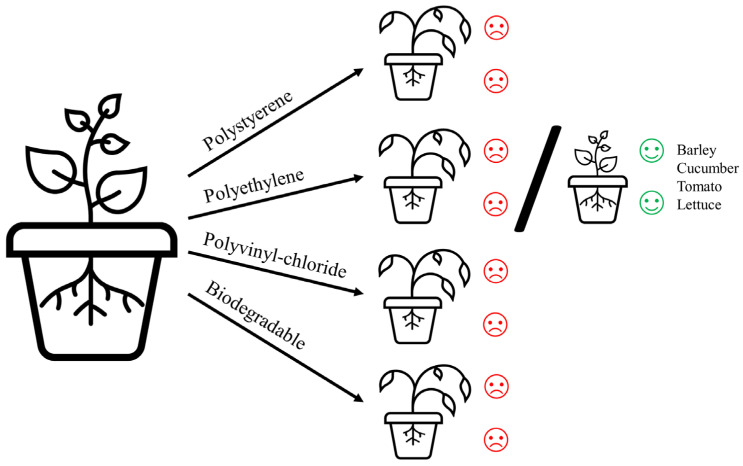
Plant growth responses to polystyrene, polyethylene, polyvinyl-chloride, and biodegradable plastics.

In summary, the effects of plastics on plant development are increasingly being investigated, and it is evident that different types of plastic can have varying impacts on plants. Consistent with previous reviews [20,59,94,95], the plant responses to plastics remain predominantly uniform. Most recent studies demonstrate growth inhibition, while only a handful of species exhibit a positive growth response, particularly in the presence of PE (Figure 1). These additions include barley [79], cucumber, tomato, and lettuce plants [50], adding to previously identified species such as *Allium fistulosum*, *T. aestivum*, *L. sativum*, *Calamagrostis epigejos*, and *Hieracium pilosella* (reviewed in [20]).

However, the aggregated results indicate that plastics, including PS, PE, PVC, and biodegradable variants, have predominantly detrimental effects on plant growth, particularly in relation to root and shoot development.

PS has consistently emerged as an inhibitor of plant growth, as evidenced by studies revealing diminished root and shoot expansion across various plant species. Likewise, PE exhibits inhibitory traits affecting crops such as maize, cucumber, and water moss, leading to compromised root and shoot proliferation. PVC’s negative impact on plant development is also evident, as demonstrated by reduced root growth in rice and plumed cockscomb plants. Despite their touted environmental friendliness, biodegradable plastics also manifest deleterious consequences for plant growth. Corn, basil, sand couch-grass, and ice plant display suppressed root and shoot development in the presence of such plastics. Moreover, PBAT and PLA hinder the germination and shoot growth of red cherry tomatoes and lettuce plants.

**Figure 2:**
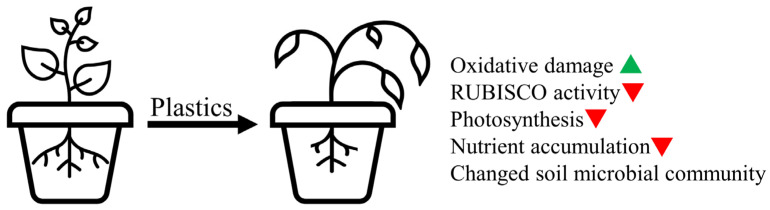
Mechanisms through which microplastics affect the growth and development of plants.

As previously summarized [20,59,94,95], the mechanisms through which microplastics affect the performance of higher plants are both diverse and intricate (Figure 2). One of the predominant outcomes triggered by MPs in plants is oxidative stress, as evidenced by the increased generation of reactive oxygen species and heightened activity of antioxidant enzymes (as reviewed by [59,94,95]). Similarly, a common feature of the results presented in the reviewed papers is the disruption of oxidative homeostasis. This alteration implies its potential involvement in shaping the plant growth responses to plastics, similar to what has been documented for numerous other abiotic stressors. Furthermore, nutrient uptake and metabolism have often been cited as plausible explanations for growth reduction (see [59,94,95]), and the most recent evidence collected in this paper also support this possibility. In addition, the effect of plastic on photosynthetic activity is well-documented [as reviewed in [59,95]], a fact confirmed by several of the recent studies collected in this paper. 

However, it is crucial to recognize that the precise processes, triggers, and outcomes of these phenomena remain unclear, highlighting the need for further research. It is also important to note that the effects of plastics on plant development may vary depending on factors such as plastic type, concentration, plant species, and environmental conditions. Nevertheless, the overall findings highlight the potential harm that plastic pollution, both conventional and biodegradable, can have on plant growth and ecosystem health.

Further research is needed in order to better understand the mechanisms underlying the negative effects of plastics on plants and to develop mitigation strategies. Most importantly, efforts should be made to reduce plastic pollution and promote the use of sustainable alternatives in order to ensure the health and sustainability of our ecosystems and ensure the continued provision of essential ecosystem services by plants. Overall, addressing plastic pollution and its impact on plant development is critical for the conservation of biodiversity, food security, and the overall well-being of our planet.

## 5. Future Directions 

Future research directions in this field involve the study of the following aspects:

- Interactions with different stressors: The interactions between plastics and other stressors, such as heavy metals or chemicals (which can be adsorbed in MPs), need to be explored further. Understanding how plastics interact with other environmental factors can provide insights into their combined effects on plant growth and development.
- Mechanisms of action: Studying the mechanisms through which plastics exert their negative effects on plants is crucial. This includes investigating how plastics are taken up by plants, their impact on cellular processes, and the disruption of plant physiology. Elucidating these mechanisms will help in designing targeted mitigation strategies.
- Species-specific responses: Different plant species may exhibit varying sensitivities to plastics. Further research should focus on a wide range of plant species in order to better understand the species-specific responses to different types of plastics and concentrations.
- Long-term effects: Most of the studies conducted so far have focused on short-term effects. It is important to investigate the long-term consequences of plastic exposure on plant growth, reproduction, and overall ecosystem health. Long-term studies can provide valuable insights into the persistence and cumulative effects of plastics on plants.
- Field studies: While many studies have been conducted under controlled laboratory conditions, field studies are necessary to assess the real-world impacts of plastics on plant development. Field experiments can consider the complex interactions of plants with their natural environment, including soil composition, nutrient availability, and microbial communities.
- Biodegradable plastics: Further research is needed to evaluate the environmental fate and potential ecological impacts of biodegradable plastics. Understanding their decomposition rates, byproducts, and effects on plant growth will help us to determine their suitability as alternatives to conventional plastics.

## Figures and Tables

**Table 2:** The effect of polyethylene-based plastics on the development of plants. “+” indicates growth induction, “0” means no change, “−” represents inhibition, while “n.d.” marks parameters not determined.

					Effect on				
MP Types	Species	Concentration	Particle Size	Medium Used	Germination	Root Growth	Shoot Growth	Notes	Reference
Polyethylene (PE)	*Zea mays* L.	0.1, 1, and 10% (*w*/*w*)	n.d.	Pot experiments	n.d.	n.d.	−	Inhibition of chlorophyll synthesis and photosynthetic rates in maize seedling leaves.	[73]
	*Z. mays*	n.d.	4 cm^2^	Pot experiment	n.d.	−	−	PE residual films had no noticeable effect on the abovementioned soil parameters.	[74]
	*Z. mays*	0.4 mg m/L	236 ± 7.4 μm and 281 ± 14 μm	In vitro experiment	n.d.	−	−	MPs may act as pollutant carriers, affecting plant physiology and transcriptomic pathways.	[75]
	*Cucumis sativus* L.	200 mg/L	13, 48, and 500 μm	Hydroponic	n.d.	−	−	Inhibited the photosynthesis of seedlings and caused lipid peroxidation.	[76]
	*Salvinia auriculata* Aubl.	1 × 1012 particles/m^3^	35.46 µm ± 18.17 µm	Hydroponic	n.d.	−	−	Effects on oxidative and nitrosative stress and changes in membrane permeability.	[77]
	*Lens culinaris* Medik	10, 50, and 100 mg/L	740–4990 nm	Optical coherence tomography (bOCT)	−	n.d.	n.d.	The inhibition of germination and seedling growth.	[78]
	*Hordeum vulgare* L.	0, 10, 100, and 1000 mg/L	790 nm–4999 nm	Petri dish experiments	0	+	+	No significant impact of PE MPs on seed germination of barley, cucumber, or tomato plants.	[79]
	*C. sativus*				0	−	+		
	*Solanum lycopersicum* L.				−	−	+		
	*Lactuca sativa* L.	1, 5, and 10% PE MPs	2–4 mm	Pot experiment	n.d.	+	n.d.	Resulted in changes in the structure and function of the soil microbial community.	[50]
High-density polyethylene (HDPE)	*H. vulgare*	2% (*w*/*w*)	1 cm × 1 cm	Glass mesocosms	n.d.	0	−	No effect on root growth but showed shoot growth inhibition.	[80]
	*Thinopyrum junceum* L.	n.d.	20 cm × 20 cm	Pot experiments	n.d.	−	−	Growth inhibition was observed.	[81]
	*Carpobrotus* sp.				n.d.	−	−		

**Table 3:** The effect of polyvinyl-chloride-based plastics on the development of plants. “0” means no change, “−” represents inhibition, while “n.d.” marks parameters not determined.

				Effect on				
Species	Concentration	Particle Size	Medium Used	Germination	Root Growth	Shoot Growth	Notes	Reference
*Zea mays* L.	0, 0.1, 1, and 10%, *w*/*w*	15 µm	Pot experiment	n.d.	−	−	SOD and CAT in leaves increased to alleviate the stress.	[83]
*Celosia argentea* L.	n.d.	0.7, 1.7, and 2.4 mm	Pot experiment	n.d.	−	−	The presence of the microplastics in the soil affected the growth of the plant significantly, some plastics affected it positively, some others negatively.	[84]
*Ipomoea batatas* L.	100–200 mg/L	6.5 µm	Hydroponic	n.d.	0	0	PVC MPs enhanced Cr(VI) accumulation and toxicity.	[85]
*Spirodela polyrhiza* L.	0, 10, 100, and 1000 mg/L	3.87 ± 3.14 μm	Open glass containers	n.d.	−	n.d.	Inhibited morphological traits, reproductive traits, and nutrient accumulation as well.	[86]
*Spirodela polyrhiza* L.	0, 10, 100, and 1000 mg/L	600 μm, 150 μm, and 13 μm	Exposure experiments	n.d.	−	n.d.	The responses of rhizosphere soil properties were reduced soil bulk density and improved soil porosity, caused by MPs.	[87]
*Oryza sativa* L.	10% (*w*/*w*)	155–180 μm	Soil incubation	n.d.	−	0	No effect on shoot growth but displayed root growth inhibition.	[88]

**Table 4:** The effect of bio-based plastics on the development of plants. “0” indicates no change, “−” represents inhibition, while “n.d.” marks parameters not determined.

MP Types	Species	Concentration	Particle Size	Medium Used	Effect on			Notes	References
					Germination	Root Growth	Shoot Growth		
Bio-Based Plastics	*Zea mays* L.	0.1%, 1%, and 10% (*w*/*w*)	-	Pot experiment	n.d.	n.d.	−	Inhibition of chlorophyll synthesis and photosynthetic rates in maize seedling leaves.	[73]
	*Z. mays*	n.d.	4 cm^2^	Pot experiment	n.d.	−	−	Soil water content, aggregate stability, inorganic nitrogen, and maize crop productivity were all influenced by BIO film residues, due to their high degradability.	[74]
	*Thinopyrum junceum* L.	n.d.	20 × 20 cm	Pot experiment	n.d.	−	−	Reduced the performance of the native species.	[81]
	*Carpobrotus* sp.				n.d.	−	−	Favored the spread of the invasive species.	
	*Ocimum basilicum* L	3.75 g of corn starch powder	5 mm	Pot experiment	n.d.	−	−	Oxidative stress was induced in the aerial part of basil plants.	[91]
	*Sorghum saccharatum* L.	0.02, 0.095, 0.48, 2.38, and 11.9% (*w*/*w*)	2.5 mm	Soil incubation	0	−	−	No effect on germination, but growth inhibition was observed.	[92]
	*Lepidium sativum* L.				0	−	−		
	*Sinapis alba* L.				0	−	−		
	*Lycopersicon esculentum* Mill.	100 and 1000 mg/kg of NPs	1 cm^2^	Pot experiment	−	n.d.	−	Tomato was more susceptible to specific BDM.	[93]
	*Lactuca sativa* L.				−	n.d.	−	Lettuce is a reliable species to identify potential BDM ecotoxicity.	
Poly(3-hydroxybutyrate-co-3-hydroxyvalerate (PHBV)	*Z. mays*	0.01, 0.1, 1, or 10%		Pot experiment	n.d.	−	−	Significant changes in the soil metabolome and microbial community, likely associated with changing function, were observed.	[90]
Polylactic acid (PLA)	*Hordeum vulgare* L.	n.d.	1 × 1 cm	Glass mesocosms	n.d.	0	−	No effect on root growth but shoot growth inhibition was observed.	[84]
	*Cucumis sativus L.*	200 mg/L	13, 48, and 500 μm	Hydroponic	n.d.	−	−	Inhibited the photosynthesis of seedlings and caused lipid peroxidation.	[76]
